# Reduced Acquisition Time [^18^F]GE-180 PET Scanning Protocol Replaces Gold-Standard Dynamic Acquisition in a Mouse Ischemic Stroke Model

**DOI:** 10.3389/fmed.2022.830020

**Published:** 2022-02-10

**Authors:** Artem Zatcepin, Steffanie Heindl, Ulrike Schillinger, Lena Kaiser, Simon Lindner, Peter Bartenstein, Anna Kopczak, Arthur Liesz, Matthias Brendel, Sibylle I. Ziegler

**Affiliations:** ^1^Department of Nuclear Medicine, University Hospital of Ludwig-Maximilians-Universität (LMU) Munich, Munich, Germany; ^2^German Center for Neurodegenerative Diseases (DZNE) Munich, Munich, Germany; ^3^Institute for Stroke and Dementia Research, University Hospital of Ludwig-Maximilians-Universität (LMU) Munich, Institute for Stroke and Dementia Research, Munich, Germany; ^4^Munich Cluster for Systems Neurology (SyNergy), Munich, Germany

**Keywords:** positron emission tomography (PET), GE-180 imaging, microglia, stroke, neuroinflammation, image-derived blood input function (IDIF), kinetic modeling, translocator protein (TSPO) imaging

## Abstract

**Aim:**

Understanding neuroinflammation after acute ischemic stroke is a crucial step on the way to an individualized post-stroke treatment. Microglia activation, an essential part of neuroinflammation, can be assessed using [^18^F]GE-180 18 kDa translocator protein positron emission tomography (TSPO-PET). However, the commonly used 60–90 min post-injection (p.i.) time window was not yet proven to be suitable for post-stroke neuroinflammation assessment. In this study, we compare semi-quantitative estimates derived from late time frames to quantitative estimates calculated using a full 0–90 min dynamic scan in a mouse photothrombotic stroke (PT) model.

**Materials and Methods:**

Six mice after PT and six sham mice were included in the study. For a half of the mice, we acquired four serial 0–90 min scans per mouse (analysis cohort) and calculated standardized uptake value ratios (SUVRs; cerebellar reference) for the PT volume of interest (VOI) in five late 10 min time frames as well as distribution volume ratios (DVRs) for the same VOI. We compared late static 10 min SUVRs and the 60–90 min time frame of the analysis cohort to the corresponding DVRs by linear fitting. The other half of the animals received a static 60–90 min scan and was used as a validation cohort. We extrapolated DVRs by using the static 60–90 min p.i. time window, which were compared to the DVRs of the analysis cohort.

**Results:**

We found high linear correlations between SUVRs and DVRs in the analysis cohort for all studied 10 min time frames, while the fits of the 60–70, 70–80, and 80–90 min p.i. time frames were the ones closest to the line of identity. For the 60–90 min time window, we observed an excellent linear correlation between SUVR and DVR regardless of the phenotype (PT *vs*. sham). The extrapolated DVRs of the validation cohort were not significantly different from the DVRs of the analysis group.

**Conclusion:**

Simplified quantification by a reference tissue ratio of the late 60–90 min p.i. [^18^F]GE-180 PET image can replace full quantification of a dynamic scan for assessment of microglial activation in the mouse PT model.

## Introduction

Stroke is the second leading cause of death worldwide and one of the leading causes of disability (1). Treatment dedicated to improving post-stroke recovery remains unresolved. One of potential targets for such treatment is post-stroke neuroinflammation due to its long-lasting nature and the presence of multiple effectors that can be targeted (2, 3). Yet, none of the so far conducted trials testing immunomodulatory therapies have been able to consistently demonstrate a beneficial function of such approaches on post-stroke recovery. There can be several possible reasons for these failures, one of which is the fact that previous studies did not take into account individual patients' differences in the extent of the neuroinflammatory response. Even though the influence of the neuroinflammatory response to stroke outcome in humans is yet to be determined, individual measures of neuroinflammation can assist in patient selection for immune interventions.

Neuroinflammation can be assessed through monitoring of microglia, the brain resident innate immune cells (4). Microglia activation is strongly correlated to the 18 kDa translocator protein (TSPO) expression level on the outer membrane of microglial mitochondria (5). However, it should be mentioned that microglia are not the only cell type expressing TSPO in the brain under disease conditions. For instance, activated astrocytes significantly contribute to the overall TSPO signal, as shown in (6) for rats with traumatic brain injury and in (7) for patients with multiple sclerosis. Multiple PET tracers targeting TSPO have been developed over the years. First-generation TSPO tracers, such as [^11^C]-I-PK11195 (8) and [^11^C]-AC-5216 (9), have low signal-to-noise ratio and high off-target binding, which prevented them from routine use. Second-generation TSPO tracers include flutriciclamide ([^18^F]GE-180), a tracer that has higher specific binding and improved signal-to-noise ratio (10, 11).

Benchmark parameters of a microglia PET quantification protocol with [^18^F]GE-180 consist of a 0–90 min post-injection (p.i.) acquisition, continuous arterial blood sampling in the first 15 min, and measurement of the tracer metabolites in the arterial blood (12). These data are required to estimate quantitative indices such as total distribution volume (V_T_) of [^18^F]GE-180 using the 2-tissue compartment model with reversible binding or the Logan plot (12, 13). However, it is not feasible to implement such a protocol in most clinical settings due to the complexity of arterial blood sampling, reduced patient comfort, and increased workload for a nuclear medicine department. In small animal studies, especially in longitudinal measurements, the arterial blood sampling is even more challenging, as, due to the limited amount of blood, it requires an external arteriovenous shunt to be installed (14).

Therefore, it is desirable to establish a simplified quantification approach that would yield a robust TSPO expression estimate without the need for the long PET acquisition and arterial blood sampling. In an experimental stroke model, we investigated the relationship between the V_T_ ratio (DVR) in the lesion area and the normal cortex tissue calculated from a long 0–90 min p.i. [^18^F]GE-180 PET scan by using Logan plot with an image-derived blood input function (IDIF) and the standardized uptake value ratio (SUVR) in the same regions estimated from one of the late 10 min time frames. We then established and validated a late 30 min time frame for simplified [^18^F]GE-180 quantification.

## Materials and Methods

### Study Design

**Figure 1:**
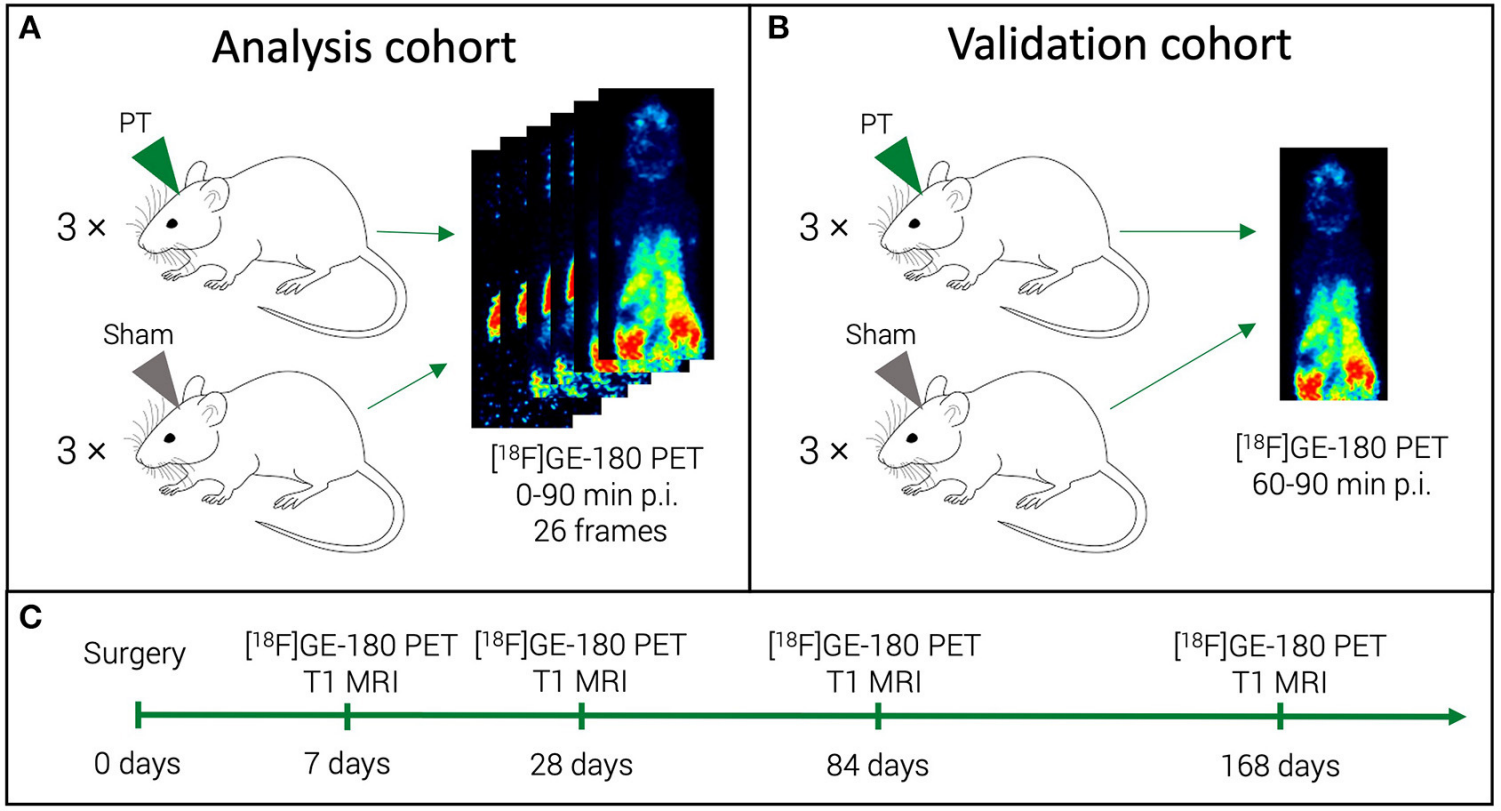
Study mice and acquired scans. For a half of the study mice (analysis cohort), we performed a dynamic [^18^F]GE-180 acquisition **(A)**. For the other half (validation cohort), a shorter scan was performed **(B)**. All the mice were scanned at four time points in a longitudinal design **(C)**.

The study was designed as a longitudinal imaging study with four TSPO-PET time points over six months (Figure 1). First, we aimed at defining a robust 30 min time frame that would provide the best semi-quantitative estimate (SUVR) for the quantitative [^18^F]GE-180 binding measure (DVR). To this end, we compared the performance of the last five 10 min frames in approximating the DVR. For this part of the study, we performed long 0–90 min p.i. PET acquisitions in half of the investigated mice (analysis cohort). Three 10 min frames that provided the best SUVR-DVR linear fits were selected and reconstructed to a corresponding 30 min frame (60–90 min p.i.) using the same list-mode data. Subsequently, we calculated the linear fit between the SUVRs for the 60–90 min frame and the DVRs. The second aim was to test if the obtained fit has a similar performance on a separate validation dataset. For the validation cohort, we performed only 60–90 min p.i. PET acquisitions. We then compared the extrapolated DVRs obtained for the validation cohort against the DVRs of the analysis cohort.

### Animals

Twelve male C57Bl/6J mice with a baseline age of 8–10 weeks were included in the study (Charles River Laboratories). All experiments were conducted in accordance with national guidelines for the use of experimental animals and all protocols were approved by the German governmental committees (Regierung von Oberbayern, Munich, Germany).

### Experimental Stroke Model

Photothrombotic (PT) stroke surgery was performed as previously described (15, 16). Briefly, the mice were anesthetized with isoflurane, delivered in a mixture of 30% O_2_ and 70% N_2_O. Mice were placed into a stereotactic frame, and body temperature was maintained at 37°C with a mouse warming pad. Dexpanthenol eye ointment was applied to both eyes. Animals received 10 μl/g body weight of 1% Rose Bengal (198250-5g; Sigma Aldrich) in saline intraperitoneally 5 min prior to anesthesia induction (5% isoflurane). A skin incision was made to expose the skull. Bregma was located, and the lesion location was marked in the left hemisphere (1.5 mm lateral and 1.0 mm rostral to bregma). Shielding was placed on the skull, allowing a 2.0 mm diameter circular light exposure over the lesion area. Ten minutes after Rose Bengal injection, the laser (25 mV) was applied to the lesion area for 17 min (Cobolt Jive 50, 561 nm; Fiber Collimation Package: 543 nm, *f* = 7.66 mm, beam diameter [d] = 1.4 mm). The sham procedure was performed in 6 out of the 12 animals (Figures 1A,B) as described above, but without laser illumination. All the animals received Buprenorphin and Carprofen as peri-surgery analgesia as well as Carprofen as post-surgery analgesia. After the surgery, the mice were placed into a warming chamber at 37°C for 20 min.

### PET/MR Imaging

The mice were scanned at 7, 28, 84, and 168 days after the PT induction (Figure 1C) using a 3T Mediso nanoScan PET/MR scanner (Mediso Ltd, Hungary) with a single-mouse imaging chamber. The mice received an intravenous injection of 18.0 ± 2.1 MBq [^18^F]GE-180 through the tail vein. For the dynamic PET imaging (3 PT and 3 sham mice), acquisition was performed from 0 to 90 min p.i. (analysis cohort) (Figure 1A). For the static PET imaging, (3 PT and 3 sham mice), the list-mode data were acquired at 60-90 min p.i. (validation cohort) (Figure 1B). A 15-min anatomical T1 MR scan was performed at 30 min after [^18^F]GE-180 injection for the validation cohort (static imaging) and after 90 min for the analysis cohort (head receive coil, matrix size 96 × 96 × 30, voxel size 0.21 × 0.24 × 0.65 mm3, repetition time 677 ms, echo time 28.56 ms, flip angle 90°). The PET field of view (FOV) included the whole mouse, while the MR FOV covered the mouse head only. The T1 image was then used to create a body-air material map for the attenuation correction of the PET data. We reconstructed the PET list-mode data within a 400–600 keV energy window using a 3D iterative algorithm (Tera-Tomo 3D, Mediso Ltd, Hungary) with the following parameters: matrix size 55 × 62 × 187 mm3, voxel size 0.3 × 0.3 × 0.3 mm3, 8 iterations, 6 subsets. When acquired dynamically (0–90 min p.i. acquisitions), the list-mode data were binned into 25 frames (6 x 10, 2 x 30 s, 3 x 1, 5 x 2, 5 x 5, 5 x 10 min). Decay and random correction were applied.

### Image Data Processing

**Figure 2:**
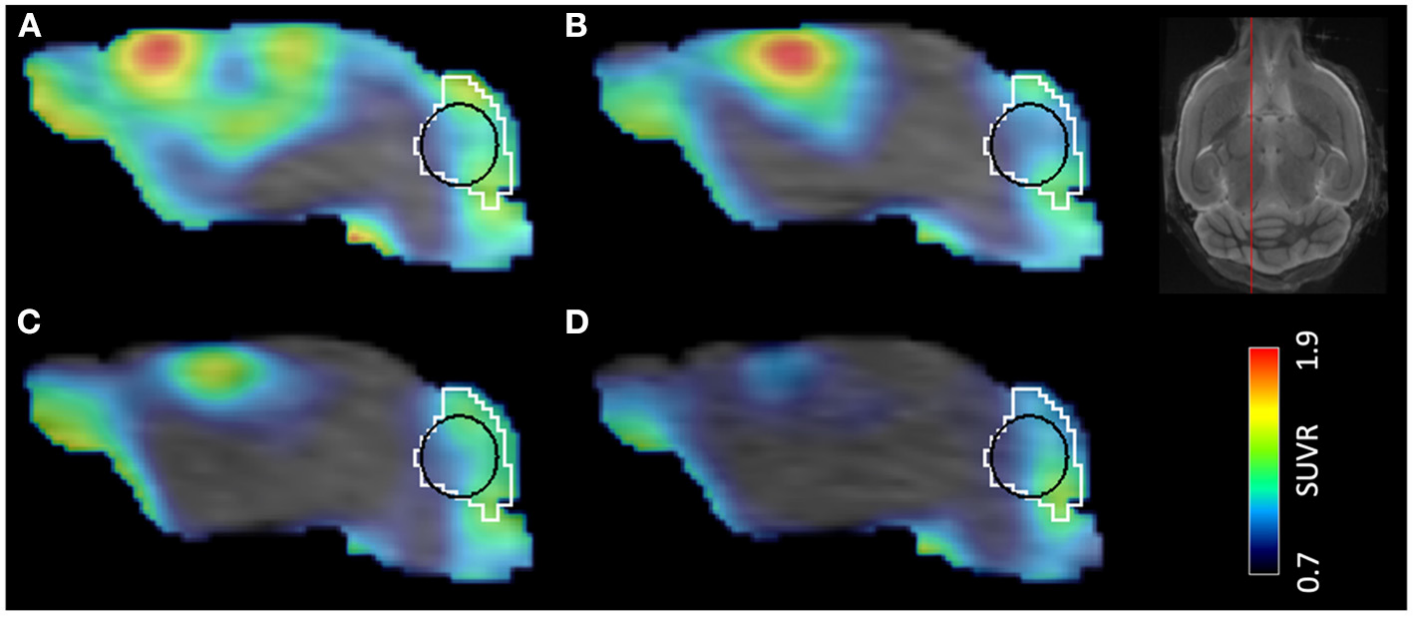
Time development of PT in a single mouse demonstrated by [^18^F]GE-180 PET/MR. Timepoints after the surgery: **(A)** 7 days, **(B)** 28 days, **(C)** 84 days, **(D)** 168 days. VOIs shown in the image: white—cerebellum, black—cerebellar white matter. The location of the sagittal slice on a template MR image is shown on the top right.

Image processing steps were performed using PMOD View and Fuse It tools (version 4.005, PMOD Technologies, Zurich, Switzerland). All T1 MR images were deformably registered to a high-resolution (0.2 × 0.2 × 0.2 mm^3^) built-in MRI template using the SPM5 procedure, and the Ma-Benveniste-Mirrione atlas (17, 18) was applied to the image to obtain the cerebellum (Figure 2) and the cortex volume of interest (VOI). Within the cerebellum VOI, we defined an elliptical volume that represented the cerebellar white matter (CBWM) (Figure 2, black line). The elastic transformation was applied to the corresponding PET image. Then, we manually segmented a predefined individual VOI in the PT area using the 7-day T1 MR image. Next, this VOI was dilated by 1 mm at all borders, for including microglia located in the perilesional area (19). Finally, we cropped this VOI by the cortex VOI, since the PT was induced in the cortex in our experiment. The resulting VOI was defined as the PT VOI in each animal.

### Total Distribution Volume Estimation

To acquire quantitative estimates of the tracer binding in the analysis cohort, an arterial input function (IF) was obtained. Since continuous arterial sampling is challenging in mice, especially for longitudinal measurements, no blood IF was available. Therefore, we estimated the V_T_ using the Logan plot (13) with an IDIF.

#### IDIF

##### Factor Analysis-Based Partial Volume Effect Correction

**Figure 3:**
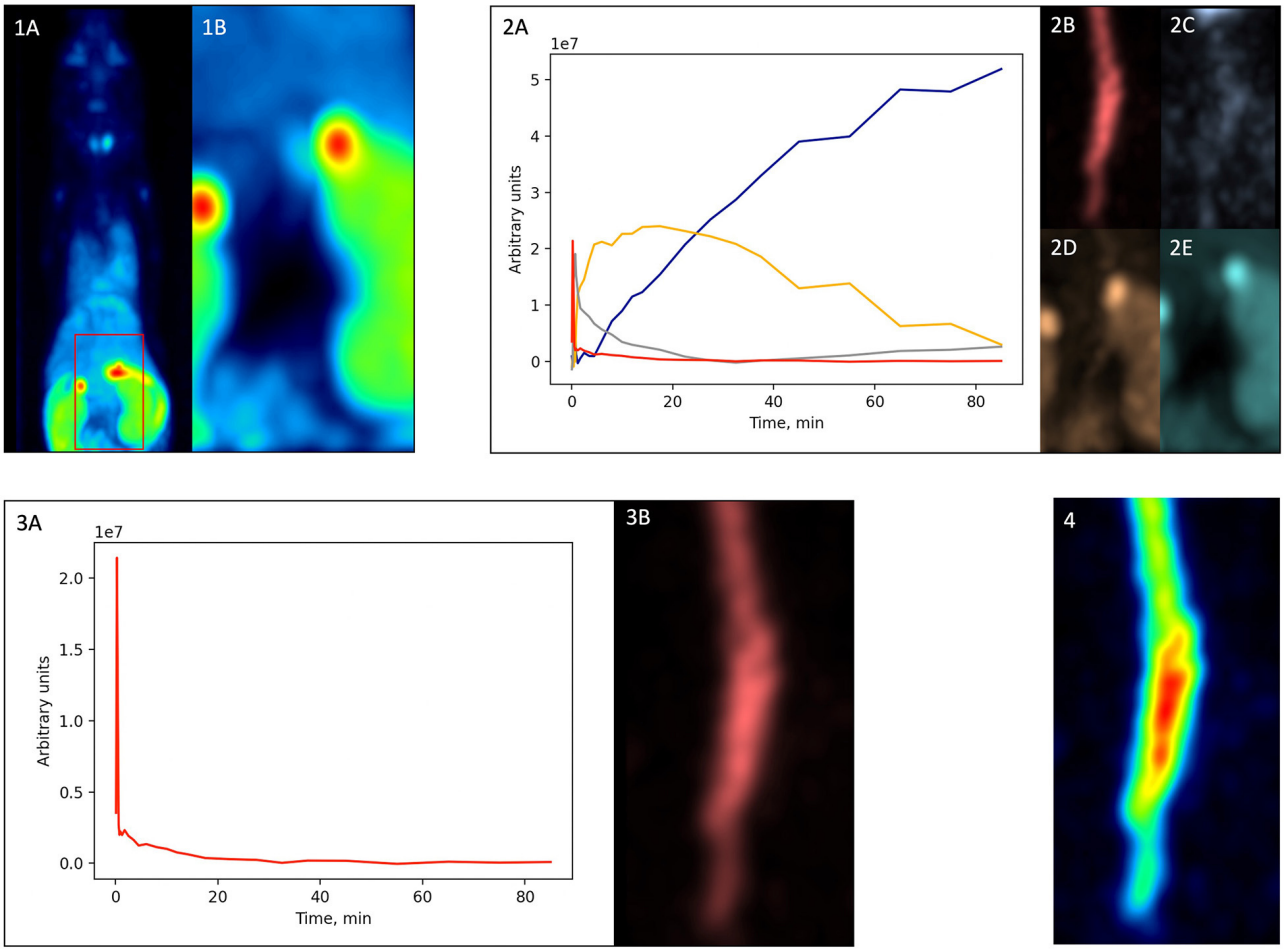
Factor analysis-based PVE correction. **(1A)**—original dynamic scan (late frame is shown); **(1B)**—vena cava region; **(2A)**—factor curves; **(2B–E)**—factor images; **(3A)**—blood factor curve *f*_1_(*t*); **(3B)—**blood factor image *a*_1_(*i*); **(4)**—spill-in corrected dynamic image $S_{i}^{*}$(t) (early frame is shown).

The IDIF was generated using the [^18^F]GE-180 activity in the vena cava. For partial volume effect (PVE) correction of the vena cava region, we performed factor analysis (FA) on a cropped image that contained the vena cava (Figure 3). FA splits the dynamic scan into several spatial distributions that represent tissues with distinct kinetics:


$$\begin{array}{l} S_{i}\left(t\right)=\;\overset{N}{\underset{k=1}{\sum }}a_{k}\left(i\right)f_{k}\left(t\right)+e_{i}\left(t\right), \end{array}$$

where *S*_*i*_(*t*) is the original dynamic scan, *a*_*k*_(*i*) is a factor image that is one of the N spatial distributions of the image *S*_*i*_(*t*), *f*_*k*_(*t*) is the curve that reflects the kinetics of that spatial distribution, *e*_*i*_(*t*) is the error, *N* is the selected number of factors, *i* represents the i^th^ voxel of the image. The used methodology was based on the approach proposed by Wimberley et al. (20) for the analysis of [^18^F]-DPA-714 mouse PET data.

First, from the original dynamic scan [Figure 3(1A)], we cropped the vena cava region based on early frames [Figure 3(1B)]. Then, we performed factor analysis using Pixies software (Apteryx, France) on this cropped image. Four factors were chosen, since this number was shown to be optimal for a TSPO tracer in mice (20). A positivity constraint was applied to both factor images and factor curves. The resulting factor curves and the corresponding factor images are shown in Figure 3(2A–E), respectively. The factor curve with the earliest peak was selected [Figure 3(2A), red]. Considering the prior knowledge that the tracer concentration in blood monotonously increases and then decreases after the peak, FA was run another time with a corresponding constraint. The resulting curve was defined as a blood curve *f*_1_(*t*) [Figure 3(3A)] and multiplied with its corresponding factor image *a*_1_(*i*) [Figure 3(3B)] using matrix multiplication,


$$\begin{array}{l} S_{i}^{*}(t)=a_{1}(i)f_{1}(t), \end{array}$$

which produced a new dynamic image $S_{i}^{*}\left(t\right)$ that included only the blood component [Figure 3(4)].

##### IDIF Estimation

We performed automatic segmentation of a VOI for the IDIF definition using a Python code based on NiBabel and NumPy libraries. The procedure consisted of taking four hottest voxels from each axial plane of the $S_{i}^{*}\left(t\right)$ image. Then, for each frame, the VOI average was calculated. This time-activity curve was then fitted using a 2-exponential linear model (21), since such a model was already successfully used in kinetic modeling of [^18^F]GE-180 PET by Fan et al. (12). The resulting curve was used as the IDIF. Plasma-to-blood ratio multiplication did not have to be performed, since the FA-derived IF represents an approximation of the plasma curve (20). No metabolite correction was applied.

#### Relationship Between DVR and SUVR

For both cohorts (analysis and validation), the SUVR in the PT VOI was calculated by normalizing the PT VOI mean uptake by the CBWM VOI mean uptake. The CBWM was selected as it had less spill-in from the skull compared to the cerebellum VOI (Figure 2). For the analysis cohort, V_T_ in the PT and in the CBWM VOI was obtained using the Logan plot (PMOD Kinetic tool), and then the PT VOI V_T_ was divided by the CBWM V_T_, resulting in a DVR value. To investigate the relationship between DVRs and SUVRs in the PT VOI of the mouse PT model as well as the sham animals, we plotted the SUVRs against the DVRs obtained from the analysis cohort using all the longitudinal scans combined. This was done for five late 10 min time frames (40–50, 50–60, 60–70, 70–80, and 80–90 min p.i.) as well as for a late 30 min frame (60–90 min p.i.). For the former we obtained the fits using both the PT and the sham mouse data combined, while for the latter we additionally performed a separate fitting for PT and sham mice. Next, we acquired 60–90 min p.i. PET data for the validation cohort and we estimated DVRs in the PT VOI by making use of the 60–90 min p.i. fits of the analysis cohort.

### Statistical Analysis

Statistical analysis was performed using Scipy and Pingouin libraries in Python. To assess the degree of linearity between the SUVR and the DVR in the PT VOI, we calculated Pearson's r (22) for values derived at all the time points from all the studied mice (24 value pairs in total) or, for the 60–90 min p.i. time frame, separately for the PT and the sham animals (12 value pairs in total in both cases). A *p*-value was calculated to assess the significance of the linear correlation. In order to determine significant over- or underestimations, a paired *t*-test was used to compare SUVR and DVR values of the analysis cohort as well as to compare extrapolated DVRs of the validation cohort and DVRs of the analysis cohort.

## Results

### Late Static SUVR Shows High Agreement With DVR for [^18^F]GE-180 TSPO-PET in an Experimental Stroke Dataset

**Figure 4:**
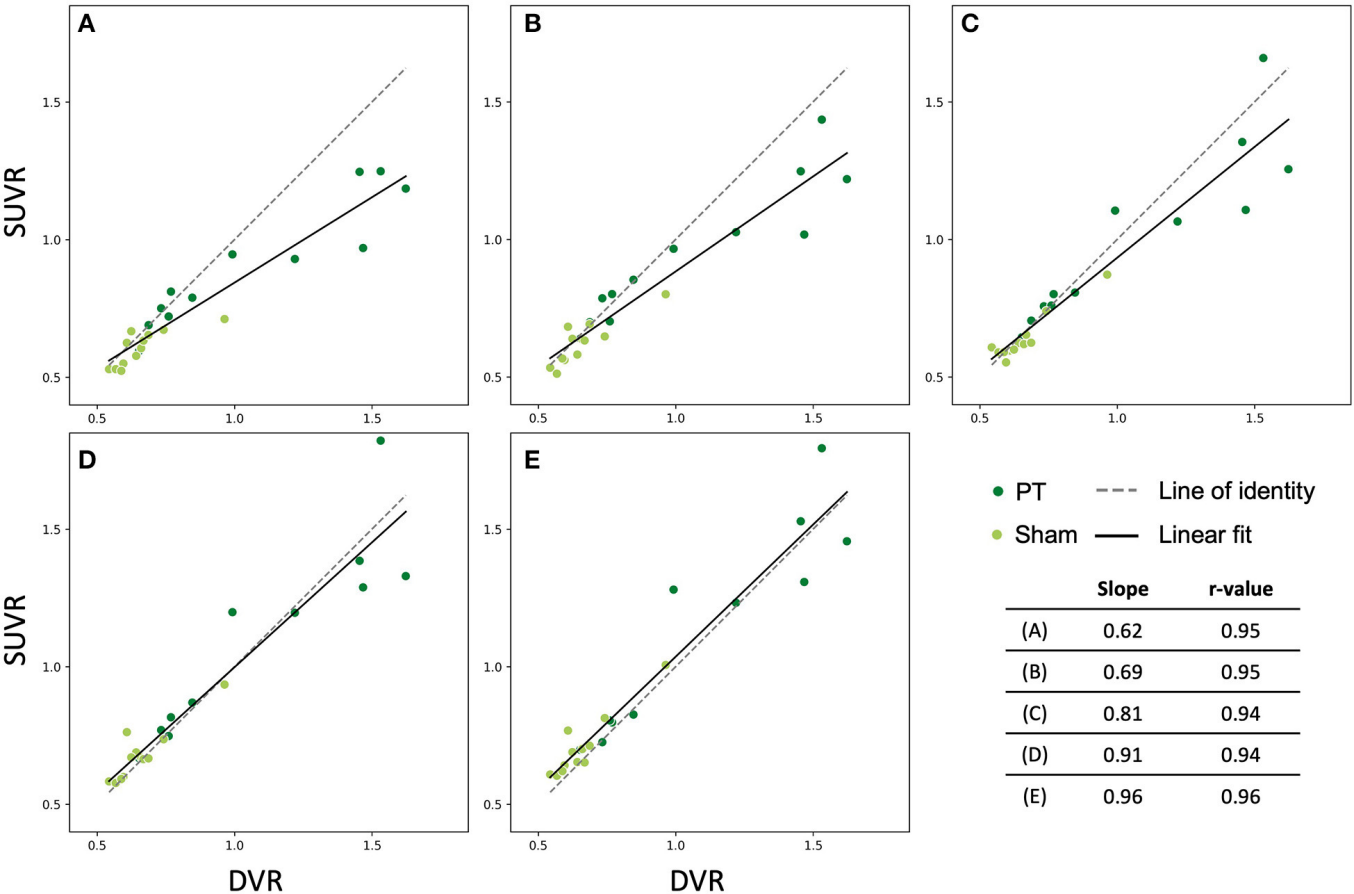
Relationship between SUVR (late 10 min frames) and DVR (0–90 min) in the lesion VOI of the studied mice (all time points). Dark green dots indicate PT mice, light green dots represent sham mice. The solid and the dashed lines are the linear fit and the line of identity, respectively. **(A)** 40–50 min frame, **(B)** 50–60 min frame, **(C)** 60–70 min frame, **(D)** 70–80 min frame, **(E)** 80–90 min frame.

We examined the late time frames (40–50, 50–60, 60–70, 70–80, and 80–90 min p.i.) from the dynamic [^18^F]GE-180 PET scans (analysis cohort) to determine which frames yield the SUVR values that approximate the DVR values in the PT VOI best. In this part of the study, we performed linear fitting on the combined data (PT and sham) (Figure 4). The fit lines of the 60–70, 70–80, and 80–90 min p.i. frames had the slope value closest to one (0.81, 0.91, and 0.96, respectively) (Figures 4C–E) and only slightly under- or overestimated the line of identity, which makes them the best approximations among the considered frames. 40−50 and 50–60 min frames significantly underestimated DVR (*p* = 0.002/*p* = 0.01). The fit parameters for the dynamic frames are summarized in Table 1.

**Table 1:** Fit parameters for the investigated frames and their corresponding *p*-values from pairwise *t*-tests with the quantification method as a within-factor (last column).

**Frame, min**	**Slope**	**Intercept**	***r*-value**	**Linear correlation *p*-value**	***t*-test *p*-value**
40–50	0.62	0.22	0.95	<10^−11^	0.002
50–60	0.69	0.19	0.95	<10^−11^	0.01
60–70	0.81	0.13	0.94	<10^−11^	0.11
70–80	0.91	0.09	0.94	<10^−11^	0.63
80–90	0.96	0.08	0.96	<10^−12^	0.05

### 60–90 min SUVR Delivers a Robust Approximation of [^18^F]GE-180 DVR in Stroke and Sham Mice

**Figure 5:**
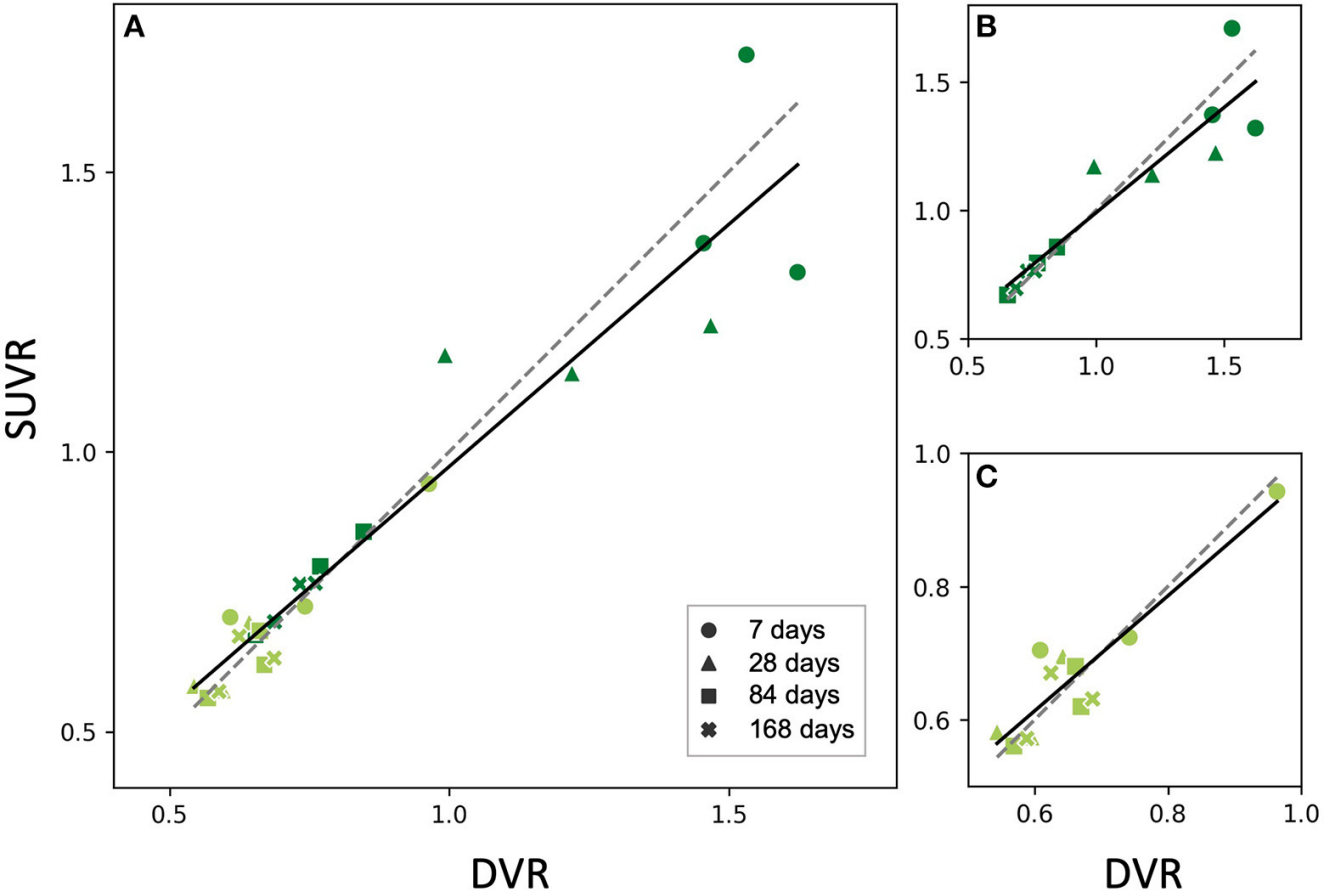
Relationship between SUVR and DVR from the static 60–90 min scan in the PT VOI of the studied mice (all time points). Dark green dots indicate PT mice, light green dots represent sham mice **(A)** PT and sham mice together, fit parameters: *SUVR* = 0.87•*DVR*+0.11, *r* = 0.95, *p* < 10^−12^, **(B)** PT mice only, fit parameters: *SUVR* = 0.82•*DVR*+0.17, *r* = 0.92, *p* < 10^−4^, **(C)** Sham mice only, fit parameters: *SUVR* = 0.86•*DVR*+0.10, *r* = 0.91, *p* = 10^−4^. All the fits (solid lines) are close to identity line (dashed). The marker shape represents the time after the surgery.

Based on this finding, we then reconstructed the list-mode data from 60 to 90 min p.i. and plotted the corresponding SUVRs against the DVRs. Figure 5 shows the relationship between SUVR and DVR when the PT and the sham mice are considered together (A) and for separate analysis of PT mice (B) and sham mice (C). A strong linear correlation was observed in all datasets (PT: *r* = 0.91, *p* < 10^−4^; sham: *r* = 0.92, *p* < 10^−4^; combined: *r* = 0.96, *p* < 10^−13^). The corresponding linear fits indicated slopes of 0.76 (PT), 0.86 (sham), and 0.84 (combined), and were located in proximity to the line of identity.

Again, we performed a paired *t*-test for comparison of SUVR and DVR as a within-factor for the 60–90 min frame. No significant differences were observed between SUVR and DVR when considering PT mice, sham mice, and the combination of both groups (*p* = 0.63, *p* = 0.69, *p* = 0.73, respectively).

### Successful Validation of DVR Extrapolation by Use of 60–90 min SUVR Fitting in an Independent Cohort

**Figure 6:**
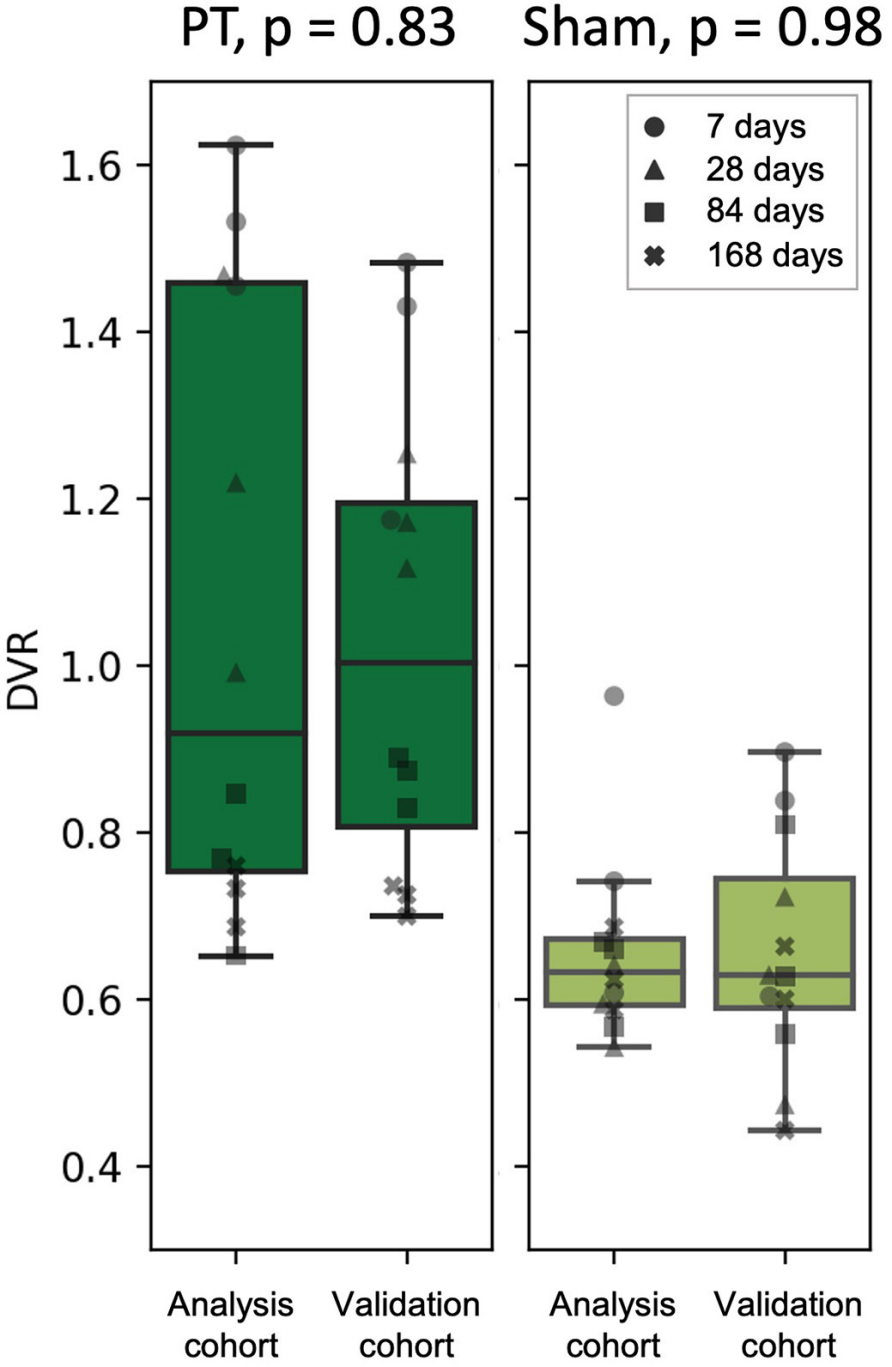
DVR calculated using Logan plot with IDIF (analysis cohort) against DVR estimated from the linear fits (validation cohort) for the PT mice **(left)** and the sham mice **(right)**. The marker shape represents the time after the surgery.

To test the prediction performance of the obtained linear fits (Figures 5B,C) on an independent dataset, we performed a PET acquisition for an additional cohort from 60 to 90 min p.i. only (validation cohort, Figure 1B). Using the PT and sham linear fits obtained for the analysis cohort as well as the PT VOI SUVRs of the validation cohort derived from the 60–90 min p.i. scans, we estimated the DVRs in the PT VOI for the validation cohort. Estimated DVRs of the validation cohort were compared to the standard of truth DVRs obtained using the Logan plot for the analysis cohort (Figure 6). For both the PT and the sham groups, we observed no significant difference between DVRs (*p* = 0.83 and *p* = 0.98, respectively). The group effect between the PT and sham mice was at a similar magnitude in the analysis (+45%, *p* = 0.0016) and the validation (+60%, *p* = 0.0004) cohort.

## Discussion

In this preclinical study, we demonstrated that semi-quantitative [^18^F]GE-180 binding in late static imaging frames can be used to substitute dynamic imaging in experimental stroke. Estimates in the PT area from all the studied time frames (40–50, 50–60, 60–70, 70–80, and 80–90 min p.i.) (SUVRs) were highly correlated to the quantitative estimates derived from a full 0–90 min p.i. scan (DVRs). Fits for the last three frames (60–70, 70–80, 80–90 min p.i.) were the ones closest to the identity line and indicated that late static imaging recapitulates DVR best. We then showed similar results for a 60–90 min p.i. scan, which contains more counts and therefore provides a more robust image, regardless of the group composition. Our findings were validated by an independent cohort that had been scanned from 60 to 90 min p.i. Importantly, DVR estimates calculated for the validation cohort using the linear fits obtained for the analysis cohort, were not significantly different from the DVRs calculated for the analysis cohort using the full-length scan. The fact that the slopes of the calculated linear fits were close to one and the corresponding intercepts were close to zero indicates that SUVR represents an adequate DVR estimate even without calibration in both the area affected by the PT as well as in the normal tissue.

We decided not to perform separate linear fits for SUVR *vs*. DVR for the individual time points (shown in Figure 1C), as by pooling the data we obtained more data points and, for the PT data, a higher range of values, which resulted in large enough variability to test the robustness of the data fits (see linear correlation *p* values in Table 1; Figure 5). As seen in Figure 5, the 7 and 28 days time points have higher spread compared to 84 and 168 days. This suggests a possibly higher error when estimating SUVR for high DVR values. Higher number of samples per time point would be needed to further evaluate this aspect.

Immunohistochemical assessment was not used in this work as we only aimed at comparing semi-quantitative to quantitative TSPO binding estimates obtained from PET. Comparing the three-dimensional distribution of these estimates to the real location of activated microglia shown by immunohistochemistry lies beyond the scope of this work and will be performed in future studies. Another objective for future investigations of stroke mechanisms in rodent models is a comparison of PT *vs*. sham animals at several individual time points. The differences between the two groups are expected to arise due to both PT-induced neuroinflammation and blood-brain barrier (BBB) disruption. Our work suggests increased TSPO signal in the PT mice at 7, 28, and 84 days after surgery (Figures 5, 6); however, more animals are required to ensure statistical significance of this conclusion.

To our knowledge, there are no large-scaled studies that performed [^18^F]GE-180 kinetic modeling with an IF in rodents. Most of the studies calculated semi-quantitative binding estimates, such as percentage of injected dose per ml (%ID/ml), standardized uptake value (SUV), or SUVR using one of late frames, e.g. 50–60 min p.i. (23, 24), 20–60 min p.i. (25), or 20–30 min p.i. (26), without comparing these estimates to a quantitative binding parameter from dynamic scans. Brendel et al. (27) performed such a comparison, but, in contrast to our work, they used a pseudo-reference tissue method, which does not require an IF, to calculate the ground truth binding estimate. The authors used forebrain SUVR from 60–90 min p.i. static scans and compared it to forebrain DVR that was derived from the reference-tissue Logan plot using 0–90 min dynamic scans and a white matter VOI as a reference region and showed an excellent correlation between the two estimates. Based on this finding, the late 60–90 min p.i. frame was used in a rat unilateral labyrinthectomy study (28). Contrary to (25), Brackhan et al. (29) compared simplified reference tissue model-derived (SRTM-derived) binding potential and %ID/ml to autoradiography results in rats and demonstrated a better correlation for the binding potential estimate. The contradictory results may be explained by the fact that [^11^C]-PK11195 used in (29) has lower specific binding compared to [^18^F]GE-180 (11). Other potential reasons could be a different species (rats *vs*. mice) and ground truth estimation method (SRTM *vs*. reference-tissue Logan plot). Our study provides evidence that the 60–90 min p.i. frame can be used in a mouse stroke model (PT) as well. Importantly, we found that frames > 60 min p.i. outperformed frames <60 min p.i. Thus, late static imaging should be used to avoid underestimation of DVR by SUVR. As of today, there are only a few studies on stroke in rodents that monitored neuroinflammation using [^18^F]GE-180. As mentioned above, Chaney et al. (23) used a 50–60 min p.i. frame to calculate SUV in a mouse ischemic stroke study. Another example of a rodent stroke study with [^18^F]GE-180 is the work performed by Boutin et al. (10). The authors used 0–60 min p.i. scans with the SRTM using the region contralateral to the stroke VOI as a reference. No comparison study with an IF has been performed, to our knowledge.

Our study provides the comparison between the DVRs and the SUVRs for the PT and healthy cortex only. Additional investigation is required to establish whether the high correlation between the two estimates stays valid for other brain tissues. Another future step could be the estimation of the relationship between quantitative and semi-quantitative binding estimates in a human stroke cohort.

One of the limitations of this study is the lack of an arterial blood IF. Instead, we took advantage of the fact that the whole mouse was always within the FOV, and we used an IDIF derived from a large vessel, the vena cava, for kinetic modeling. An IDIF might not precisely represent the tracer blood peak due to the finite length of the first frames (10 s in this study) and is prone to PVE. However, an effort was made in this work to reduce the PVE in the IDIF VOI by using FA. Additionally, PT might lead to systemic inflammation and increase the number of circulatory inflammatory cells, which would make the IDIF plateau in the PT mice higher than in the sham mice. FA solves this issue, since the factor curve with the earliest peak, which was selected as the blood curve, does not include the signal coming from specific binding in the whole blood and therefore in its shape resembles the plasma curve as shown in presaturation experiments (20). The IDIF eliminates some innate sources of error of the arterial IF, such as delay, dispersion between the arterial tree and the sampling site, and blood counter-scanner cross-calibration. An IDIF methodology was already successfully implemented for mouse [^18^F]GE-180 PET data by Xiang et al. (30), where we obtained an IDIF from a bilateral VOI placed in both carotid arteries to estimate V_T_ images. The IDIF estimated using the vena cava tends to overestimate the real blood peak value, so one might consider correcting it for bolus dispersion. However, Lanz et al. (31) showed that the dispersion-corrected IDIF did not outperform the raw IDIF in a similar kinetic model when using rat FDG-PET data.

Another limitation of our study is the lack of plasma metabolite correction for [^18^F]GE-180. For mice, no literature on metabolite data is available to our knowledge. Human studies suggest a high parent fraction concentration in the plasma compared to the metabolites (around 70% of the intact [^18^F]GE-180 remaining at 90 min p.i.) (12, 32, 33). Based on these data, not correcting for metabolites might have only a moderate effect on the binding estimates. Moreover, in this work, ratios of V_T_ were used instead of the absolute V_T_, which is likely to reduce the influence of the plasma metabolites.

Of note, there are several issues when considering TSPO as a marker for activated microglia in stroke. Weber et al. (34) were able to demonstrate increased BBB permeability in the mouse PT model during 3 weeks after the surgery. This suggests that peripheral immune cells possibly enter the lesion area. These cells cannot be distinguished from microglia as they also express TSPO. Some studies also suggest significant differences in microglia and macrophages activation between rodents and humans (5). Additional studies are required to confirm the results of our work in humans after ischemic stroke.

[^18^F]GE-180 is known to have relatively low brain uptake and high blood retention. However, to our knowledge, there are no alternative TSPO tracers with properties significantly superior to those of [^18^F]GE-180. Additionally, [^18^F]GE-180 offers several advantages relevant for this study, such as significant correlation between [^18^F]GE-180 PET and Iba1 in mice (27, 35) and the ability to detect neuroinflammation after cerebral ischemia in mice (23). Additionally, as mentioned before, [^18^F]GE-180 has higher binding potential compared to [^11^C](R)-PK11195 (11) and high [^18^F]GE-180 parent fraction is observed even at late time points (12, 32). In a recent work, Kaiser et al. (36) demonstrated high [^18^F]GE-180 PET signal in glioma subvolumes without MRI contrast enhancement (Gd-BOPTA) in a voxel-wise analysis and detected a large distance between the hotspots delineated in contrast-enhanced MRI and [^18^F]GE-180 PET, which proves the specificity of [^18^F]GE-180 PET signal and its relative independence of BBB disruption.

In conclusion, our results prove that static 60–90 min p.i. [^18^F]GE-180 PET can be a substitute for dynamic imaging with IF when assessing post-stroke neuroinflammation in the PT mouse model.

## Data Availability Statement

The raw data supporting the conclusions of this article will be made available by the authors, without undue reservation.

## Ethics Statement

The animal study was reviewed and approved by German Governmental Committees (Regierung von Oberbayern, Munich, Germany).

## Author Contributions

AZ: data analysis, concept, and manuscript writing. SH and US: data acquisition. LK: data analysis. SL: tracer production. PB: concept. AK: proofreading. AL: proofreading and concept. MB and SZ: concept and manuscript writing. All authors contributed to the article and approved the submitted version.

## Funding

This project was supported by the Deutsche Forschungsgemeinschaft (DFG, German Research Foundation) under Germany's Excellence Strategy (EXC 2145 SyNergy – ID 390857198) and through FOR 2879 (project number 428668490).

## Conflict of Interest

The authors declare that the research was conducted in the absence of any commercial or financial relationships that could be construed as a potential conflict of interest.

## Publisher's Note

All claims expressed in this article are solely those of the authors and do not necessarily represent those of their affiliated organizations, or those of the publisher, the editors and the reviewers. Any product that may be evaluated in this article, or claim that may be made by its manufacturer, is not guaranteed or endorsed by the publisher.
